# 

**Published:** 1928

**Authors:** 


### The Medical Library of the University of Bristol,

WITH WHICH IS INCORPORATED

**The Library of the Bristol Medico-Chirurgical Society.**

*The following donations have been received since the publication of the List in September*, 1928.

*December*, 1928.

| | |
|---|---|
| Aberdeen University (1) .. .. .. | 1 volume. |
| B. M. A. Secretary (2) .. .. .. .. | 1 ,, |
| Mr. C. Osmond Bodman (3) .. .. .. | 2 volumes. |
| British Empire Cancer Campaign (4) .. | 1 volume. |
| Dental Board of the United Kingdom (5) .. | 1 ,, |
| Professor E. Fawcett (6) .. .. .. | 1 ,, |
| Professor E. W. Hey Groves (7) .. .. | 15 volumes. |
| Dr. Bernard Hollander (8) .. .. .. | 1 volume. |
| London School of Hygiene and Tropical Medicine (9) .. .. .. .. .. | 1 ,, |
| Michell Clarke Memorial Fund (10) .. .. | 2 volumes. |
| Rockefeller Foundation (11) .. .. .. | 1 volume. |
| Sir Humphry D. Rolleston, Bart. (12) .. | 2 volumes. |
| University of Otago Medical School (13) .. | 1 volume. |

Unbound periodicals have also been received from Dr. Carey F. Coombs, Dr. C. Corfield and Professor E. W. Hey Groves.

### THE ONE HUNDRED AND TWENTY-SIXTH LIST OF BOOKS.

The figures in round brackets refer to the figures after the names of the donors. The books to which no such figures are attached have either been bought from the Library Fund or received through the *Journal*.

| | | | |
|---|---|---|---|
| **Aschner, B., and Engelmann, G.** | *Konstitutionspathologie in der Orthopädie* | (7) | 1928 |
| **Barnard, W. G.** | *Elementary Pathological Histology* | | 1928 |
| **Briggs, I. G.** | *How to Start in General Practice* | | 1928 |
| **Brockbank, E. M., and Ramsbottom, A.** | *Clinical Examination of the Lungs* | 2nd Ed. | 1928 |
| **Broderick, F. W.** | *Dental Medicine* | | 1928 |
| **Cathcart, C. W., and Hartley, J. N. J.** | *Requisites and Methods in Surgery* | | 1928 |
| **Da Costa, J. C.** | *Modern Surgery* | (7) | 1925 |
| **Douthwaite, A. H.** | *The Injection Treatment of Varicose Veins* | 3rd Ed. | 1928 |
| **Dowden, J. W.** | *Clinical Surgery* | | 1928 |
| **Erlacher, P. J.** | *Die Technik des Orthopädischen Eingriffs* | (7) | 1928 |
| **Fairbairn, J. S.** | *Gynæcology with Obstetrics* | 2nd Ed. | 1928 |
| **Fenwick, P. C. C.** | *Insomnia and Drug Addiction* | | 1928 |
| **Field, L. R.** | *Primary Neoplasms of the Urinary Bladder* | | 1928 |
| **Forgue, E.** | *An der Schwelle der Chirurgie* | (7) | 1928 |
| *Gastro-Intestinal Diseases.* (James Mackenzie Institute) | | | 1928 |
| **Haehl, R.** | *Samuel Hahnemann* | 2 Vols. (3) | 1928 |
| **Haire, N.** | *The Comparative Value of Current Contraception Methods* | | 1928 |
| **Ham, B. B.** | *Handbook of Sanitary Law* | 10th Ed. | 1928 |
| **Hanbold, H. A.** | *Principles and Practice of Surgery* | 2 Vols. (7) | 1921 |
| **Hollander, B.** | *In Commemoration of F. J. Gall* | (8) | 1928 |
| **Horder, Sir T., and Gow, A. E.** | *Essentials of Medical Diagnosis* | | 1928 |
| **Hutchison, R.** | *Diagnosis, Prognosis and Treatment* | | 1928 |
| *International Conference on Cancer*, London, 1928 | | (4) | 1928 |
| **Johnston, W.** | *Roll of Commissioned Officers in the Medical Services of the British Army*, 1727–1898 | (1) | 1917 |
| **Kidd, B.** | *Science of Power* | (7) | 1918 |
| **König, E.** | *Die Körpereigene freie Fascienverpflanzung* | (7) | 1928 |
| **Kopp, J. G.** | *L'ostéosynthèse dans le mal de Pott* | (7) | — |
| **Leveuf, J., etc.** | *Fractures et Luxations* | (7) | 1925 |
| **Lindsay, D. M.** | *Obstetric and Gynæcological Cases* | | 1928 |
| **Martindale and Westcott** | *Extra Pharmacopœia.* Vol. I. | 19th Ed. | 1928 |
| **Melchior, E.** | *Nachbehandlung nach Chirurgischen Eingriffen* | (7) | 1928 |
| **Miles, W. E.** | *Cancer of the Rectum* | (7) | 1926 |
| **Nesfield, V.** | *Deafness and its Alleviation* | | 1928 |
| *Nouveau Traité de Médécine.* Vol. XVI. | | (10) | 1928 |
| **Osler and McCrae** | *Modern Medicine*, 3rd Ed., *Index to the Work* | (10) | 1928 |
| **Pearse, J.** | *Public Health Services* | (6) | 1928 |
| *Radiology and Radiotherapy* | | (5) | 1298 |
| *Rheumatic Diseases. The Bath Conference* | | | 1928 |
| *The Robert Jones Birthday Book* | | | 1928 |

| | | |
|---|---|---|
| **Roberts, J. B., and Kelly, J. A.** | *Fractures* .. .. .. .. .. 2nd Ed. (7) | 1921 |
| **Rolleston, Sir H.** .. | *Aspects of Age, Life and Disease*.. .. .. (12) | 1928 |
| **Rolleston, Sir H.** .. | *Cardio-Vascular Diseases since Harvey's Discovery* (12) | 1928 |
| **Schwalbe, J.** (Ed.) .. | *Diagnostiche und Therapeutische Irrtümer und deren Verhütung. Chirurgie. Heft* 9 .. (7) | 1928 |
| **Simms, W.** .. .. .. .. | *Manual of Dental Prosthetics* .. .. .. .. .. | 1928 |
| **Singer, C.** .. .. .. .. | *Short History of Medicine* .. .. .. .. .. .. | 1928 |
| **Symes, J. O.** .. .. | *Erythema Nodosum* .. .. .. .. .. .. .. .. .. .. | 1928 |
| **Taylor, F. L.** .. .. | *Crawfurd W. Long* .. .. .. .. .. .. .. .. .. .. | 1928 |
| **Watson, J. K.** .. .. | *Handbook for Nurses* .. .. .. .. .. 8th Ed. | 1928 |
| **Weisz, E.** .. .. .. .. | *Diagnostik mit Freiem Auge, 3te Aufl* .. (7) | 1928 |
| **Wilson, A. G.** .. .. | *Orthodontics* .. .. .. .. .. .. .. .. .. .. .. .. | 1928 |
| **Wolff, E.** .. .. .. .. | *A Shorter Anatomy* .. .. .. .. .. .. .. .. .. .. | 1928 |
| **Wright, S.** .. .. .. .. | *Applied Physiology* .. .. .. .. .. .. 2nd Ed. | 1928 |

TRANSACTIONS, REPORTS, JOURNALS, ETC.

| | |
|---|---|
| B.M.A. Annual Handbook, 1928–29 .. .. .. .. .. .. .. .. .. (2) | 1928 |
| London School of Hygiene and Tropical Medicine. Collected Addresses Vol. 4 .. .. .. .. .. .. .. .. .. .. .. .. .. .. .. .. .. .. .. (9) | 1928 |
| Medical Directory, 1929 .. .. .. .. .. .. .. .. .. .. .. .. .. .. .. .. | 1928 |
| Methods and Problems of Medical Education. Series 10 .. .. .. (11) | 1928 |
| Proceedings of the University of Otago Medical School. No. 5 .. .. (13) | 1928 |
| Progressive Medicine. September, 1928 .. .. .. .. .. .. .. .. .. .. .. | 1928 |
| Quarterly Cumulative Index Medicus. (3) .. .. .. .. .. .. .. .. .. .. | 1928 |
| St. Bartholomew's Hospital Reports. Vol. 61 .. .. .. .. .. .. .. .. | 1928 |
| St. Thomas's Hospital Reports. Vol. 50 .. .. .. .. .. .. .. .. .. .. | 1928 |
| Surgical Clinics of North America. August and October, 1928 .. .. | 1928 |
| Transactions of the College of Physicians of Philadelphia. Vol. 49 .. .. | 1927 |

### Local Medical Notes.

EXAMINATION RESULTS.

### University of Bristol.

M.D.—C. B. Perry, M.B., Ch.B., M.R.C.P., A. M. Critchley, M.B., Ch.B.

M.B., Ch.B.—*Supplementary First Examination. Pass:* J. F. H. Eagles, Winifred M. Hill, D. S. Prichard. *In Chemistry and Biology only:* Mary G. Thomas, S. P. N. Williams.

M.B., Ch.B.—*Final Examination* (*Part I.*), *including Forensic Medicine and Toxicology. Pass:* J. F. Coates (with distinction in Materia Medica, Pharmacy, Pharmacology and Therapeutics, and Forensic Medicine and Toxicology), Eveline M. D. M. Collinson, H. F. G. Corfield (with distinction in Forensic Medicine and Toxicology), J. L. W. Davies, J. J. J. Giraldi, R. D. Jones, G. F. Langley (with distinction in Materia Medica, Pharmacy, Pharmacology and Therapeutics), R. A. Lattey, R. P. Lucas, J. E. Newton, S. C. Wake. *In Part I. only :* U. M. Hopkins, W. L. Sleight, H. M. Strover.

M.B., CH.B.—*Final Examination* (*Part II.*). *Pass with Second Class Honours :* A. A. Dowling (with distinction in Obstetrics), H. D. Pyke (with distinction in Obstetrics). *Pass :* G. R. Fells (with distinction in Public Health), Ethel M. Redman. *In Group II.* (*completing Examination*) *:* Phoebe C. Vine.

B.D.S.—*Third Examination. Pass : In Dental Metallurgy and Dental Mechanics only :* W. Forsyth. *In Dental Materia Medica only :* Muriel E. S. Cosh.

L.D.S.—*Supplementary Preliminary Science Examination. Pass : In Biology only* (*completing Examination*) *:* G. N. Minifie.

L.D.S. — *Second Professional Examination. Pass : In Dental Mechanics and Metallurgy* (*completing Examination*) *:* E. R. Carpenter. *In Dental Mechanics* (*completing Examination*) *:* I. W. Williams. *In Dental Materia Medica* (*completing Examination*) *:* J. D. Galloway. *In Dental Materia Medica only :* N. Miller.

L.D.S.—*Final Professional Examination. Pass :* Barbara J. Shapland.

**University of London.**—M.B., B.S., Group I., *Medicine.*—Doreen G. C. Nixon, M.R.C.S., L.R.C.P.

**Conjoint Board.**—F.R.C.S.—*Primary Examination :* E. F. King, M.B., Ch.B. (Bristol), D.O.M.S., N. C. Tanner.

M.R.C.S., L.R.C.P.—*Pre-medical :* Emina M. Hamdy (*in Physics*).

M.R.C.S. L.R.C.P. — *First Examination. Part II.* (*Pharmacy and Materia Medica*) *:* W. L. Sleight, J. L. W. Davies.

M.R.C.S., L.R.C.P.—*Final Examination.*—R. A. S. Cory, J. V. Lucas (*in Midwifery only*), R. J. Krogh (*in Midwifery only*), N. D. Gerrish (*in Midwifery only*), V. E. N. Allen (*in Surgery, completing Examination*).

L.D.S., R.C.S.—*First Professional Examination. Part I.* (*Dental Mechanics*) *:* J. K. Noot, Q. A. Davies, E. S. Edwards, W. F. G. Harvey, J. J. Hinton, J. P. Price.

L.D.S., R.C.S.—*First Professional Examination. Part II.* (*Dental Metallurgy*) *:* D. R. Jones, W. R. R. Newton, L. Symonds, K. V. M. Taylor-Milton, C. A. G. Evans.

L.D.S., R.C.S.—*First Professional Examination. Part III.* (*Anatomy and Physiology*) *:* G. E. Chadd, E. R. Dowland, E. S. Edwards, W. U. Harwood, R. E. Morgan, A. M. Watson, J. H. White.

L.D.S., R.C.S.—*First Professional Examination. Part III.*(*b*). (*Dental Anatomy*) *:* Q. A. Davies, J. D. Sellers.

**Society of Apothecaries.**—L.S.A.—*Surgery :* L. P. Gregory.

---

**The Annual Medical Dinner** of the Bristol Medical School was held on Thursday, December 13th, at the University Union (Victoria Rooms). Past and present students, practitioners in Bristol and the neighbourhood, and members of the hospital staffs to the number of 158 met under the presidency of Dr. Newman Neild, F.R.C.P., the guest of the evening being Dr. R. A. Young, C.B.E., F.R.C.P., Senior Physician to the Middlesex Hospital. In proposing the King's health the President made reference to His Majesty's serious illness, and the anxious hope with which the whole nation waited for news of further improvement in his condition. After the loyal toast had been drunk the President proposed the health of Dr. Young, and this was received with loud acclamation. Dr. Young, in responding, proposed "The Bristol Medical School," and Lieut.-Colonel G. S. Parkinson, D.S.O., R.A.M.C. (retired), and Mr. Hambly replied. Dr. Carey Coombs proposed the toast of the President, to which Dr. Newman Neild replied.

After the tables had been cleared a most enjoyable dance took place, at which the wives of the "honoraries" of the B.R.I. and B.G.H. were the hostesses.

### Index.

Blachford, J. V.—The early diagnosis and treatment of Insanity, 253.

Books, Reviews of—
Abdominal Surgery of Children, The. —L. E. Barrington-Ward, 147.
Anatomy, A Shorter.—E. Wolff, 309.
Aplastic Anæmia, Acute.—A. H. Smith, 220.
Applied Physiology.—S. Wright, 313.
Barker, J. E.—Chronic Constipation, 146.
Barrington - Ward, L. E. — The Abdominal Surgery of Children, 147.
Barwell, H.—Diseases of the Larynx, 223.
Birth Control, Practical.—E. A. Hornibrook, 149.
Brend, W. A.—Handbook of Medical Jurisprudence, 222.
Briggs, J. G.—How to start in General Practice, 219.
Brockbank, E. M.—Clinical examination of the Lungs, 220.
Broderick, F. W.—Dental Medicine, 317.
Broderick, R. A. — Dental Bacteriology, 149.
Buxton, J. L. D.—Dental Pathology, 150.
Buxton, J. L. D.—Dental Surgery, 150.
Cathcart, C. W. and Hartley, J. N. J. —Requisites and Methods in Surgery, 316.
Chronic Constipation.—J. E. Barker, 146.
Clark, C. A.—Dental Radiography, 67.
Clinical Examination of the Lungs.— E. M. Brockbank, 220.
Clinical Surgery.—J. W. Dowden, 222.
Colon, Tonic Hardening of the.— T. S. Wilson, 61.
Communicable Diseases in Schools.— Medical Officers of Schools Association, 223.
Crawford, A. M.—Materia Medica for Nurses, 150.
Crookshank, F. G.—Diagnosis and Spiritual Healing, 143.

Books, Reviews of (continued)—
Cumberbatch, E. P.—Diathermy, 66.
Cushing, H.—The Meningiomas, 311.
Dental Bacteriology. — R. A. Broderick, 149
Dental Medicine.—F. W. Broderick, 317.
Dental Pathology.—J. L. D. Buxton, 150.
Dental Radiography.—C. A. Clark, 67.
Dental Surgery.—J. L. D. Buxton, 150.
Dermatological Neuroses. — W. J. O'Donovan, 65.
Diagnosis, Prognosis and Treatment. —R. Hutchison, 309.
Diagnosis and Spiritual Healing.— F. G. Crookshank, 143.
Diathermy.—E. P. Cumberbatch, 66.
Disease, the Nature of (Pt. II).— J. E. R. McDonagh, 60.
Diseases of the Larynx.—H. Barwell, 223.
Diseases and Disorders of Childhood. —G. F. Still, 144.
Douthwaite, A. H.—The Injection Treatment of Varicose Veins, 64, 312.
Dowden, J. W.—Clinical Surgery, 222.
Erythema Nodosum.—J. O. Symes, 314.
Exposures of Long Bones.—A. K. Henry, 67.
Extra Pharmacopœia, Vol I., ed. 19 —Martindale and Westcott, 317.
Fascial Grafting in Principle and Practice.—H. C. Orrin, 221.
Fatalism or Freedom.—C. J. Herrick, 59.
Fenwick, P. C. C.—Insomnia and Drug Addiction, 314.
Fifield, L. R.—Neoplasms of the Bladder, 312.
Fitzwilliams, D. C. L.—The Tongue and its Diseases, 62.
Gastro - intestinal Diseases. — D. Waterston, ed., 314.
General Practice, How to start in.— J. G. Briggs, 219.
Goodall, E. W.—A Textbook of Infectious Diseases, 145.

BOOKS, REVIEWS OF (continued)—
Ham, B. B.—Handbook of Sanitary Law, 310.
Ham, C. I. and Short, A. R.—Synopsis of Physiology, 311.
Handbook for Nurses, A.—J. K. Watson, 317.
Handbook of Sanitary Law.—B. B. Ham, 310.
Hartley, J. N. J. and Cathcart, C. W.—Requisites and Methods in Surgery, 316.
Henry, A. K.—Exposures of Long Bones, etc., 67.
Herrick, C. J.—Fatalism or Freedom, 59.
History of Medicine, A Short.—C. Singer, 219.
Hollos, J.—Tuberculous Intoxications, 221.
Horder, Sir T.—The Essentials of Medical Diagnosis, 310.
Hornibrook, E. A.—Practical Birth Control, 149.
Hutchison, R.—Some Principles of Diagnosis, Prognosis and Treatment, 309.
Idiosyncrasies.—Sir H. Rolleston, 63.
Infectious Diseases.—E. W. Goodall, 145.
Insomnia and Drug Addiction.—P. C. C. Fenwick, 314.
Llewellyn, L. J. — Aspects of Rheumatism and Gout, 63.
Long, Crawford W., and the discovery of Ether Anæsthesia.—Frances L. Taylor, 223.
Lungs, Diseases of the.—F. E. Tylecote, 64.
Mackenzie, D.—Diseases of Nose, Throat and Ear, 148.
McDonagh, J. E. R.—The Nature of Disease (Pt. II.), 60.
Malignant Diseases, the Surgical Treatment of.—Sir H. J. Waring, 147.
Martindale and Westcott.—Extra Pharmacopœia, Vol. 1., ed. 19, 317.
Materia Medica for Nurses.—A. M. Crawford, 150.
Medical Annual, The, 1928, 224.
Medical Diagnosis, The Essentials of.—Sir T. Horder, 310.
Medical Jurisprudence and Toxicology, Handbook of.—W. A. Brend, 222.
Medical Officers of Schools Association. — Prevention of Curable Diseases in Schools, 223.
Meningiomas, The.—H. Cushing, 311.
Mental Disorders.—H. J. Norman, 146.

BOOKS, REVIEWS OF (continued)—
Mitchell, T. W. — Problems in Psychopathology, 66.
Neoplasms of the Bladder.—L. R. Fifield, 312.
Norman, H. J.—Mental Disorders, 146.
Nose, Throat and Ear, Diseases of the.—W. S. Syme, 143.
Nose, Throat and Ear, Diseases of.—D. Mackenzie, 148.
O'Donovan, W. J.—Dermatological Neuroses, 65.
Orrin, H. C.—Fascial Grafting, 221.
Orthodontics.—A. G. Wilson, 313.
Pneumothorax and Surgical Treatment of Pulmonary Tuberculosis.—C. Riviere, 62.
Post-mortem Appearances.—J. M. Ross, 149.
Proceedings of Bath Conference, May, 1928.—Rheumatic Diseases, 312.
Psychopathology, Problems in.—T. W. Mitchell, 66.
Requisites and Methods in Surgery.—C. W. Cathcart and J. N. J. Hartley, 316.
Rheumatic Diseases. — Conference Proceedings, Bath, May, 1928, 312.
Rheumatism and Gout, Aspects of.—L. J. Llewellyn, 63.
Riviere, C. — Pneumothorax and Surgical Treatment of Pulmonary Tuberculosis, 62.
Robert Jones Birthday Volume, The.—By Various Authors, 315.
Rolleston, Sir H.—Idiosyncrasies, 63.
Ross, J. M.—Post-mortem Appearances, 149.
Short, A. R. and Ham, C. I.—Synopsis of Physiology, 311.
Sinclair, M. — The Thomas Splint, 59.
Singer, C.—A Short History of Medicine, 219.
Smith, A. H. — Acute Aplastic Anæmia, 220.
Social Factors in Medical Progress.—B. J. Stern, 65.
Stern, B. J.—Social Factors in Medical Progress, 65.
Still, G. F.—Common Disorders and Diseases of Childhood, 144.
Surgical "Dont's" and "Do's."—C. H. Whiteford, 145.
Syme, W. S.—Handbook of Diseases of the Nose, Throat and Ear, 143.
Symes, J. O.—Erythema Nodosum, 314.
Synopsis of Physiology.—A. R. Short and C. I. Ham, 311.

BOOKS, REVIEWS OF (continued)—
Taylor, Frances L.—Crawford W. Long and the Discovery of Ether Anæsthesia, 223.
Thomas Splint, The.—M. Sinclair, 59.
Tongue, The, and its Diseases.—D. C. L. Fitzwilliam, 62.
Tuberculous Intoxications.—J. Hollos, 221.
Tylecote, F. E.—Diseases of the Lungs, 64.
Varicose Veins, the Injection Treatment of.—A. H. Douthwaite, 64, 312.
Waring, Sir H. J.—The Surgical Treatment of Malignant Disease, 147.
Waterston, D. ed.—Gastro-intestinal Diseases, 314.
Watson, J. K.—A Handbook for Nurses, 317.
Whiteford, C. H.—Surgical " Dont's " and " Do's," 145.
Wilson, A. G.—Orthodontics, 313.
Wilson, T. S.—Tonic Hardening of the Colon, 61.
Wolff, E.—A Shorter Anatomy, 309.
Wright, S.—Applied Physiology, 313.

Bristol Medico-Chirurgical Society, 77.
Bristol Medico - Chirurgical Society, Past Presidents, 78.
Brown, M. Forrester.—Treatment of Infantile Paralysis of the Lower Limb, 295.
Bush, G. B.—Redundant Colon, 181.

Cancer, Chemotherapeutic Researches on.—J. A. Murray, 217.
Carleton, H. H.—Problems in the Treatment of Hæmoptysis, 39.
Carwardine. T. — The Signs and Symptoms of Duodenal Ulcer, 279.
Cerebro-Spinal Fluid, Value of the Chloride Content of.—H. Rogers, 197.
Chemotherapeutic Researches on Cancer. J. A. Murray, 217.
Chitty, H.—Recent Experiences of Intussusception, 52.
Clarke, R. C.—The Medical Treatment of Duodenal Ulcer, 289.
Colloidal State of Matter, The.—F. Francis, 177.
Colon, Redundant.—G. B. Bush, 181.
Colston Research Society, 153.
Critchley, A. M.—Ocular Manifestations following Encephalitis Lethargica, 113.

Davies, Dr. D. S., Presentation to, 151.
Delusions, The Reality of (Long Fox Lecture, 1927).—H. Devine, 19.
Dermatology, Some Problems in.—W. K. Wills, 1.
Dermatology, The Comparative Value of Radiation Therapy in.—E. Dore, 85.
Devine, H.—The Reality of Delusions (Long Fox Lecture, 1927), 19.
DISCUSSIONS :
Gastric and Duodenal Ulcer in Infants.—J. A. Nixon, 72. Discussed by D. C. Rayner, 73; T. Carwardine, 73; H. J. D. Smythe, 73; C. F. Coombs, 73.
Tuberculosis Infection in the Fascial and Nasopharyngeal Tonsils of Children.—G R. Scarff, 73. Discussed by W. K. Wills, H. H. Carleton, A. J. M. Wright, E. Watson-Williams, A. W. Adams, A. E. Claremont, J. A. Nixon, G. Hadfield, W. A. Jackman, A. Alexander, 74.
Dore, E. — Radiation Therapy in Dermatology, 85.
Duodenal Ulcer, The Signs and Symptoms of.—T. Carwardine, 279.
Duodenal Ulcer, The Medical Treatment of.—R. C. Clarke, 289.

Editorial Notes.—68, 151, 225, 319.
Encephalitis Lethargica, Ocular Manifestations following. — A. M. Critchley, 113.
Erythema Nodosum, The Relationship of to Tuberculosis (Long Fox Lecture, 1928).—J. O. Symes, 233.
Erythema Nodosum, A Note on the Pathology of.—A. C. Fisher, 137.
Examination Results.—76, 163, 231, 326

Fisher, A. C.—A Note on the Pathology of Erythema Nodosum, 137.
Fisher, A. G. T.—Indications for Manipulative Surgery, 165.
Francis, F.—The Colloidal State of Matter, 177.

Gastric and Duodenal Ulcer in Infants.—J. A. Nixon, 72.
Groves, E. W. Hey.—Peri-arterial Sympathectomy, 273.

Hæmoptysis, Problems in the Treatment of.—H. H. Carleton, 39.
Haldane, The Right Hon. the Viscount.—Obituary, 227.
Harty, J. P. I.—Obituary, 155.
Heart Failure, The Evidences of.—P. Christine Vine, 97.
Hospital Ball, 320.

Infantile Paralysis of the Lower Limb, Treatment of.—M. Forrester Brown, 295.
Insanity, The Early Diagnosis and Treatment of.—J. V. Blachford, 253.
Intussusception, Recent Experiences of.—H. Chitty, 52.

King's Health. The, 319.

List of Subscribers to the Bristol Medico-Chirurgical Journal, 79.
Local Medical Notes.—76, 162, 231, 326.
Long Fox Memorial Lecture, The.—1927, The Reality of Delusions.—H. Devine, 19. 1928, The Relationship of Erythema Nodosum to Tuberculosis.—J. O. Symes, 233.
Lymphocytosis of Infection, The.—T. Wilson-Smith, 187.

Manipulative Surgery, Indications for.—A. G. T. Fisher, 165.
Medical Library of the University of Bristol, The.—75, 159, 230, 324.
Meetings of Societies—
  Bristol and Bath Surgical Club, 158.
  Bristol Medico-Chirurgical Society, 71, 228.
  British Medical Association—Bath and Bristol Branch, 229.
  Clinical Society of Bath, 322.
Meningococcal Septicæmia. — C. B. Perry, 207.
Mitral Stenosis with Auricular Fibrillation.—K. H. Pridie, 125.
Murray, J. A. — Chemotherapeutic Researches on Cancer, 217.

Nixon, J. A.—Gastric and Duodenal Ulcer in Infants, 72.

Obituary—
  The Right Hon. the Viscount Haldane, 227.
  J. P. I. Harty, 155.
  F. J. Wethered, 321.
  Sir Dawson Williams, 68.
  Sir George Alfred Wills, Bart., 154.
Ocular Manifestations following. Encephalitis Lethargica. — A. M. Critchley, 113.

Peri-Arterial Sympathectomy.—E. W. Hey Groves, 273.
Perry, C. B. — Meningococcal Septicæmia, 207.
Presidential Address—Some Developments in Dermatology during the Last Thirty Years.—W. K. Wills, 1.
Pridie, K. H.—A Case of Mitral Stenosis with Auricular Fibrillation, 125.
Pyke, H. D.—Sugar Tolerance Tests, 129.

Radiation Therapy in Dermatology.—E. Dore, 85.
Redundant Colon.—G. B. Bush, 181.
Rheumatic Diseases, Conference on, 70.
Rogers, H.—Value of the Chloride Content of the Cerebro-Spinal Fluid, 197.
Royal Medico-Psychological Association, The, 70.
Royal Society of Medicine, 152.
Royal Visit to Winford, The, 225.

Scarff, G. R.—Tuberculous Infection in the Fascial and Nasopharyngeal Tonsils of Children, 73.
Students' Number of the Bristol Medico-Chirurgical Journal, 153.
Sugar Tolerance Tests.—H. D. Pyke, 129.
Symes, J. O.—The Relationship of Erythema Nodosum to Tuberculosis (Long Fox Lecture, 1928), 233.
Sympathectomy, Peri-Arterial.—E. W. Hey Groves, 273.

Tuberculous Infection in the Fascial and Nasopharyngeal Tonsils of Children.—G. R. Scarff, 73.

Vine, P. Christine.—The Evidences of Heart Failure, 97.

Wethered, F. J.—Obituary, 321.
Williams, Sir Dawson.—Obituary, 68.
Wills, Sir George Alfred, Bart.—Obituary, 154.
Wills, W. K.—Some Developments in Dermatology, 1.
Wilson-Smith, T.—The Lymphocytosis of Infection, 187.
Winford, The New Country Hospital at, 69.
Winford, The Royal Visit to, 225.

J. W. ARROWSMITH LTD., BRISTOL

### Publications Received.

From JOHN BALE, SONS AND DANIELSSON, LTD.:

*Short Essays on Medical Topics.* By C. O. HAWTHORNE, M.D. 1928. Price 4s. 6d. net.

From CASSELL AND CO. LTD.:

*The Essentials of Medical Diagnosis.* By SIR THOMAS HORDER, Bart., K.C.V.O., M.D., F.R.C.P. Lond., and A. E. GOW, M.D., F.R.C.P. Lond. 1928. Price 16s. net.

From WILLIAM HEINEMANN (MEDICAL BOOKS) LTD.:

*The Treatment of Varicose Veins by Intravenous Injections.* By J. D. P. MCLATCHIE, M.D. C.M. 1928. Price 3s. 6d. net.

From "THE LANCET" LTD.:

*Guy's Hospital Reports.* October, 1928. 12s. 6d. net. Annual subscription two guineas net, post free.

From H. K. LEWIS AND CO. LTD.:

*A Patient's Manual of Diabetes.* By HERBERT W. MOXON, B.A., M.R.C.S., L.R.C.P. 1929 Price 6s. net.

*Chronic (non-tuberculous) Arthritis.* By A. G. TIMBRELL FISHER, F.R.C.S. (Eng.). 1928. Price 25s. net.

*Elementary Pathological Histology.* By W. G. BARNARD. 1928. Price 7s. 6d. net.

From FABER AND GWYER, LTD.:

*A Handbook for Nurses.* By J. K. WATSON, M.D. Edin., late Capt., R.A.M.C. Eighth Edition. 1928. Price 8s. 6d. net.

From DR. J. F. MONTAGUE, 30 East 40th St., New York City:

*Taking the Doctor's Pulse and The Possibilities of Medical Movies.* By J. F. MONTAGUE, M.D., F.A.C.S. 1928. For private distribution. Nominal price $1.00.

From OXFORD UNIVERSITY PRESS:

*Applied Physiology.* By SAMSON WRIGHT, M.D., M.R.C.P. With Introduction by SWALE VINCENT. Second Edition. 1928. Price 18s. net.

*Lipiodol in the Diagnosis of Thoracid Disease.* By F. G. CHANDLER, M.A., M.D. (Cantab.), F.R.C.P. (Lond.) and W. BURTON WOOD, M.A., M.D. (Cantab.), M.R.C.P. (Lond.) 1928. Price 10s. 6d. net.

From KEGAN PAUL, TRENCH, TRUBNER AND CO. LTD.

*Aspects of Age, Life and Disease.* By SIR HUMPHRY ROLLESTON, Bart., K.C.B., M.D., Hon. D.Sc., D.C.L., LL.D. 1928. Price 10s. 6d. net.

*Autolycus, or the Future for Miscreant Youth.* By R. G. GORDON, M.D., D.Sc., F.R.C.P. (Edin.). 1928. 2s. 6d. net.

*Epidemiology Old and New.* By SIR WILLIAM HAMER, M.A., M.D., F.R.C.P. 1928. Price 9s. net.

*Mescal. The "Divine" Plant and its Psychological Effects.* By HEINRICH KLÜVER, Ph.D. With Introduction by MACDONALD CRITCHLEY, M.D., M.R.C.P. 1928. Price 2s. 6d.

*Mirror-Writing.* By MACDONALD CRITCHLEY, M.D., M.R.C.P. 1928. Price 2s. 6d.

*The Troubled Conscience.* By CHARLES BLONDEL. 1928. Price 2s. 6d. net.

From SHERRATT AND HUGHES, Manchester:

*Cancer and Cancer Research.* Compiled by a Scientific Committee of the Liverpool Medica Research Organisation. 1928. Price 1s. 6d.

From JOHN WRIGHT AND SONS LTD.:

*An Index of Symptomatology.* Ed. by H. LETHEBY TIDY, M.A., M.D. (Oxon.), F.R.C.P. (Lond.). 1928. Price £2 2s. 0d. net.

*Erythema Nodosum.* By J. ODERY SYMES, M.D. 1928. Price 5s. net.

*Index of Differential Diagnosis of Main Symptoms.* By Various Writers. Edited by HERBERT FRENCH, C.B.E., M.A., M.D. (Oxon.), F.R.C.P. (Lond.). Fourth Edition. 1928. Price 63s. net.

*The British Journal of Surgery.* Vol. xvi. No. 62. October, 1928. 12s. 6d. net.

### Bristol Medico-Chirurgical Journal.

Published Quarterly (royal 8vo reduced) under the auspices of the Bristol Medico-Chirurgical Society.

*An excellent means of advertising*

The only Medical Journal published in the West of England, and has an extensive circulation not only amongst the Medical men of Bristol, but also amongst those of the adjoining Counties and South Wales, and in exchange goes to all parts of the world.

**Terms for Advertisements:**

**Whole Page, £2 2s., Half Page, £1 5s., Quarter Page, 15s. per issue.**

***PRICES FOR SPECIAL POSITIONS ON APPLICATION.***

J. W. ARROWSMITH LTD.,

**Advertising Agents,**

11 Quay Street, BRISTOL.

